# On the cytokine/chemokine network during *Plasmodium vivax* malaria: new insights to understand the disease

**DOI:** 10.1186/s12936-017-1683-5

**Published:** 2017-01-24

**Authors:** Natália Satchiko Hojo-Souza, Dhelio Batista Pereira, Fernanda Sumika Hojo de Souza, Tiago Antônio de Oliveira Mendes, Mariana Santos Cardoso, Mauro Shugiro Tada, Graziela Maria Zanini, Daniella Castanheira Bartholomeu, Ricardo Toshio Fujiwara, Lilian Lacerda Bueno

**Affiliations:** 1 Departamento de Parasitologia, Instituto de Ciências Biológicas, Universidade Federal de Minas Gerais, Belo Horizonte, Minas Gerais Brazil; 2 Centro de Pesquisa em Medicina Tropical, Porto, Velho, Rondônia Brazil; 3 Departamento de Ciência da Computação, Universidade Federal de São João del-Rei, São João del-Rei, Minas Gerais Brazil; 4 Departamento de Bioquímica e Biologia Molecular, Universidade Federal de Viçosa, Viçosa, Minas Gerais Brazil; 5 Instituto de Pesquisa Clínica Evandro Chagas, Fundação Oswaldo Cruz, Rio de Janeiro, Rio de Janeiro Brazil

**Keywords:** *Plasmodium vivax*, Malaria, Cytokines, Chemokines

## Abstract

**Background:**

The clinical outcome of malaria depends on the delicate balance between pro-inflammatory and immunomodulatory cytokine responses triggered during infection. Despite the numerous reports on characterization of plasma levels of cytokines/chemokines, there is no consensus on the profile of these mediators during blood stage malaria. The identification of acute phase biomarkers might contribute to a better understanding of the disease, allowing the use of more effective therapeutic approaches to prevent the progression towards severe disease. In the present study, the plasma levels of cytokines and chemokines and their association with parasitaemia and number of previous malaria episodes were evaluated in *Plasmodium vivax*-infected patients during acute and convalescence phase, as well as in healthy donors.

**Methods:**

Samples of plasma were obtained from peripheral blood samples from four different groups: *P. vivax*-infected, *P. vivax*-treated, endemic control and malaria-naïve control. The cytokine (IL-6, IL-10, IL-17, IL-27, TGF-β, IFN-γ and TNF) and chemokine (MCP-1/CCL2, IP-10/CXCL10 and RANTES/CCL5) plasma levels were measured by CBA or ELISA. The network analysis was performed using Spearman correlation coefficient.

**Results:**

*Plasmodium vivax* infection induced a pro-inflammatory response driven by IL-6 and IL-17 associated with an immunomodulatory profile mediated by IL-10 and TGF-β. In addition, a reduction was observed of IFN-γ plasma levels in *P. vivax* group. A lower level of IL-27 was observed in endemic control group in comparison to malaria-naïve control group. No significant results were found for IL-12p40 and TNF. It was also observed that *P. vivax* infection promoted higher levels of MCP-1/CCL2 and IP-10/CXCL10 and lower levels of RANTES/CCL5. The plasma level of IL-10 was elevated in patients with high parasitaemia and with more than five previous malaria episodes. Furthermore, association profile between cytokine and chemokine levels were observed by correlation network analysis indicating signature patterns associated with different parasitaemia levels.

**Conclusions:**

The *P. vivax* infection triggers a balanced immune response mediated by IL-6 and MCP-1/CCL2, which is modulated by IL-10. In addition, the results indicated that IL-10 plasma levels are influenced by parasitaemia and number of previous malaria episodes.

## Background

Malaria is caused by a protozoan of the genus *Plasmodium* and is responsible for high morbidity rates (besides the cases of mortality, especially among children), resulting in serious impact on the socio-economic development in endemic regions. In the Brazilian Amazon region, *Plasmodium vivax* is the main species causing malaria, being responsible for 82% of the cases [1]. Malaria is a complex disease involving genetic factors inherent to the parasite and to the host, geographical and environmental aspects that favour its occurrence and the difficulty of its eradication [2].

During infection, both the antibody-mediated and the cell-mediated immunity play an important role for achieving clinical immunity [3]. Several studies suggest that successful resolution of malaria infection depends on the ability of the host in inducing adequate levels of pro-inflammatory and regulatory cytokines during key stages of the infection. Thus, the fine-tuning between inflammatory and anti-inflammatory response appears to be a determinant factor in the clinical outcome of the disease [3–5].

Although the mechanisms involved in host immunological response during human malaria are still poorly understood, accumulating data suggest that malaria infection induces pro-inflammatory cytokines that eliminate the parasite or promote the removal of red blood cells infected by the parasite [6]. This response is suppressed in turn by anti-inflammatory cytokines, and the clearance of remaining parasites as well as the prevention of recrudescence or re-infection, are mediated by anti-parasite antibodies [6]. However, depending on factors such as genetic variability of host and parasite, age, number of infections and co-infections, the inflammatory response may be unregulated and, if excessive, it can lead to immunopathology.

Several studies have focused on the profile of plasma cytokines and chemokines in vivax malaria infection, comparing the repertoire of cytokines/chemokines elicited between *P. vivax* and *Plasmodium falciparum* infection [7–11] and further association with disease severity, determined by clinical symptoms, or immunological profile after treatment with anti-malarial drugs [8–15]. Overall the profile of cytokine/chemokine production is still contradictory due to differences in study population, degree of endemicity in the region, among other factors, requiring further investigations.

The characterization of immune responses elicited during vivax malaria and correlations with the clinical symptoms can reveal important aspects for understanding the pathogenesis of the disease and provide insights for the development of more effective vaccines and even new therapeutic approaches. Because of this, the plasma levels of pro- and anti-inflammatory cytokines and some chemokines were measured in this work during the acute phase of *P. vivax* naturally infected individuals. Samples were also obtained of some patients after anti-malarial drugs treatment to detect possible changes in the host immunological response after treatment and to identify biomarkers of active infection. Finally, analyses of correlation among cytokines/chemokines levels, degree of parasitaemia and number of infections were performed to evaluate whether variations in clinical manifestations are associated with activated or suppressed cytokine/chemokine networks.

## Methods

### Study participants and blood sample collection

*Plasmodium vivax* naturally infected individuals with uncomplicated symptomatic malaria (*P. vivax* group, n = 75), *P.vivax* naturally infected individuals after 25 days of treatment with chloroquine and primaquine (*P. vivax*-treated group, n = 10) and non-infected subjects with previous episodes of malaria (endemic control group, n = 10) were recruited at the Centro de Pesquisa em Medicina Tropical (Porto Velho, Rondônia, Brazil). In addition, 15 healthy donors (malaria-naïve control group) with no previous malaria exposure were recruited from a non-endemic area (Belo Horizonte, Minas Gerais, Brazil). The demographic, parasitological and clinical parameters of the subjects are shown in Table 1. The parasitological demonstration of *P. vivax* infection was performed by well-trained microscopists from the Centro de Pesquisa em Medicina Tropical using thick smears and it was further confirmed by nested polymerase chain reaction (PCR) as previously described [16]. Peripheral venous blood was collected in heparin-containing tubes and centrifuged to obtain plasma. Samples were stored at −80 °C until performing the cytokine and chemokine assays.

**Table 1:** Demographic, parasitological and symptomatological parameters of the study population

Parameters	Value for group			
	Control (n = 15)	Endemic Control (n = 10)	*P. vivax* (n = 75)	*P. vivax*-treated (n = 10)
Age [median(range)]	27 (19–35)	38 (21–49)	37 (20–80)	42 (21–50)
Gender [n(%)]				
Male	10 (66.7)	6 (60.0)	57 (76.0)	6 (60.0)
Female	5 (33.3)	4 (40.0)	18 (24.0)	4 (40.0)
Parasitaemia (parasites/mm^3^), [n = (%)]				
≤500	–	–	34 (45.3)	–
501–10,000	–	–	26 (34.7)	–
10,001–100,000	–	–	8 (10.7)	–
Without information	–	–	7 (9.3)	–
Nº of previous malaria episodes [n(%)]				
First malaria	–	3 (30.0)	10 (13.3)	1 (10.0)
≤5	–	2 (20.0)	24 (32)	2 (20.0)
>5	–	5 (50.0)	33 (44.0)	6 (60.0)
Without information		0 (0.0)	8 (10.7)	1 (10.0)
Symptoms [n(%)]				
Fever	–	–	66 (97.1)	–
Headache	–	–	66 (97.1)	–
Myalgia	–	–	61 (89.7)	–
Chills	–	–	60 (88.2)	–
Sweating	–	–	51 (75.0)	–
Arthralgia	–	–	49 (72.1)	–
Nausea	–	–	36 (52.9)	–
Vomiting	–	–	20 (29.4)	–

The parasitaemia and symptoms in the *P. vivax*-treated group refers to acute phase. The n for symptoms were: *P. vivax* group (n = 68) and *P. vivax*-treated group (n = 8)

### Cytokine and chemokine plasma levels assays

Measurements of IL-6, IL-10, IL-17, MCP-1/CCL2, RANTES/CCL5 and IP-10/IP-10/CXCL10 in the plasma samples were conducted using cytometric bead assay (CBA) (BD Biosciences, USA) according to manufacturer’s instructions. The data were collected using a FACSCan flow cytometer (BD Biosciences, USA) and the results were analysed in FCAP Array software (Soft Flow). The limit of detection for each assay was: IL-6 = 2.4 pg/mL, IL-10 = 4.5 pg/mL, IL-17 = 18.9 pg/mL, MCP-1/CCL2 = 2.7 pg/mL, RANTES/CCL5 = 1.0 pg/mL and IP-10/CXCL10 = 2.8 pg/mL. The upper-range limits of detection for the assays were 5000.0 pg/mL for chemokines (MCP-1/CCL2, RANTES/CCL5 and IP-10/CXCL10), and 2500.0 pg/mL for cytokines (IL-6, IL-10 and IL-17) detection. Dilution of samples was performed whenever necessary to ensure the obtained values fell within the range of the generated standard curve.

Enzyme-linked immunosorbent assay (ELISA) was performed for the measurement of IL-12p40, IL-27, IFN-γ, TNF and TGF-β (R&D Systems, USA), according to manufacturer’s instructions. Biotin-labeled antibodies were used for detection and the assay was revealed with streptavidin-HRP (Amersham Biosciences, USA) using OPD (o-Phenylenediamine dihydrochloride) substrate system (Sigma, USA). The colorimetric reaction was read using an automated ELISA microplate reader (Versamax, Molecular Devices, USA) at 492 nm. The cytokine concentration was calculated from the standard curve using seven-parameter curve fitting software (SOFTmaxPro 5.3, Molecular Devices). The limit of detection for each assay was 156.0 pg/mL for IL-27, 62.5 pg/mL for IL-12p40, 31.2 pg/mL for TGF-β, and15.6 pg/mL for IFN-γ and TNF. The upper-range limits of detection for the assays were 10,000.0 pg/mL for IL-27, 4000.0 pg/mL for IL-12p40, 2000.0 pg/mL for TGF-β, and 1000.0 pg/mL for IFN-γ and TNF. Dilution of samples was performed whenever necessary to ensure the obtained values fell within the range of the generated standard curve.

### Statistical analysis

Statistical analyses were conducted using the Prism software 5.0 for Windows (GraphPad Inc, USA). Initially, Grubb’s test was applied to detect possible outliers and the Kolmogorov-Smirnoff test was used to verify the data distribution. Comparisons among groups were performed using Kruskal–Wallis test followed by Dunn’s Post-hoc test or Mann–Whitney U test. The paired *t* test and Wilcoxon test were also applied, according to data distribution. Statistical differences were considered significant when p values were less or equal to 0.05. Results were corrected for multiple comparisons as needed.

Correlation networks were generated by the analysis of relationship among cytokine and chemokine plasma level datasets. Initially, pair-wise Spearman correlation coefficients were calculated using a scientific computing library (SciPy) and python programming language. Along with the Spearman rank-order correlation coefficient, the p value to test for non-correlation was evaluated using p ≤ 0.05 as a cut-off. The correlation strength was separated into three ranges: weak (0.2 ≤ r < 0.5), moderate (0.5 ≤ r < 0.7) and strong (0.7 ≤ r ≤ 1.0).

## Results

### *Plasmodium vivax* infection triggers a marked pro-inflammatory cytokine response

**Fig. 1:**
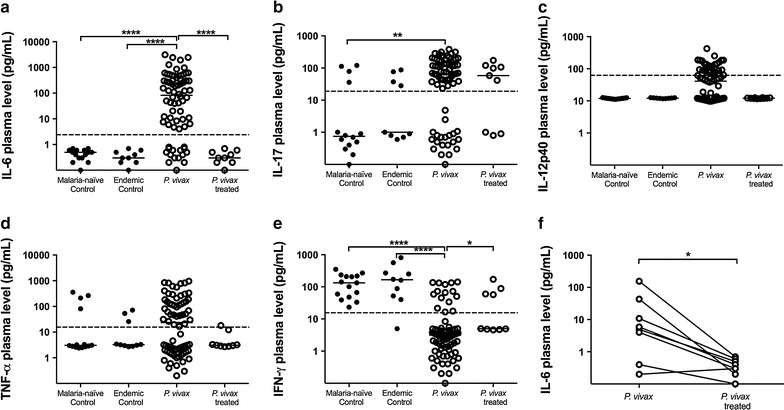
Inflammatory cytokine plasma levels. Comparative analysis of IL-6 (**a**), IL-17 (**b**), IL-12p40 (**c**), TNF (**d**) and IFN-γ production among malaria-naïve control group (n = 15), endemic control group (n = 10), *P. vivax* group (n = 75) and *P. vivax*-treated group (n = 10) were performed using Kruskal–Wallis test followed by Dunn Post-hoc. The *dotted lines* (—) represent the detection limit of the assay. IL-6 levels in acute-phase and convalescence period from *P. vivax*-infected patients (**e**) was compared between *P. vivax* group (n = 10) and *P. vivax*-treated group using Paired *t*-test or Wilcoxon test, according to data distribution. A p value <0.05 was considered significant. *p < 0.05, **p < 0.01, ***p < 0.001, ****p < 0.0001

The circulating levels of pro-inflammatory cytokines such as IL-6, IL-17, IL-12p40, and TNF were prominent in *P. vivax* naturally infected individuals when compared to plasma levels observed in other groups (malaria-naïve, endemic and *P. vivax*-treated) (Fig. 1a–d), although significant differences to control groups were observed only for IL-6 and IL-17 (p < 0.0001 and p = 0.0051, respectively) (Fig. 1a, b). Conversely, the *P. vivax* infection resulted in a significant reduction of IFN-γ plasma levels when compared to control groups (p < 0.0001) (Fig. 1e), with circulating levels of IFN-γ detected in only 20% of the samples from *P. vivax* group.

After treatment, production of IL-6, IL-12p40, TNF and IFN-γ were restored to baseline levels (p < 0.001 for IL-6 and p < 0.05 for IFN-γ, only), in contrast to plasma levels of IL-17, which were similar to those presented by infected individuals before treatment. In addition, it was also observed a significant decrease of IL-6 after treatment during a paired analysis (Fig. 1f).

### Higher levels of IL-10 and TGF-β were also induced during *Plasmodium vivax* infection

**Fig. 2:**
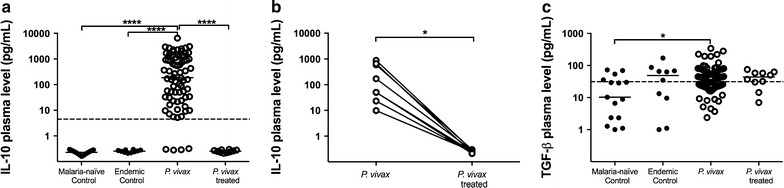
Regulatory cytokine plasma levels. Comparative analysis of IL-10 (a total production; b paired analysis between infected and treated individuals) and TGF-β among malaria-naïve control group (n = 15), endemic control group (n = 10), *P. vivax* group (n = 75) and *P. vivax*-treated group (n = 10) were performed using Kruskal–Wallis test followed by Dunn Post-hoc. A p value <0.05 was considered significant. The *dotted lines* (—) represent the detection limit of the assay. Paired analysis was performed between *P. vivax* group (n = 10) and *P. vivax*-treated group using Paired *t*-test or Wilcoxon test, according to data distribution. *p < 0.05, **p < 0.01, ***p < 0.001, ****p < 0.0001

Production of IL-10 was only observed in the *P. vivax* group, being present in 94.4% of the plasma samples (median = 186.1 pg/mL). After treatment, the IL-10 level returned to basal levels, equivalent to levels observed in control groups (p < 0.001) (Fig. 2a). Similar result was observed for the paired analysis comparing the cytokine levels before and after treatment (Fig. 2b). Moreover, samples from *P. vivax* group presented significant higher levels of TGF-β (median = 42.8 pg/mL) when compared to malaria-naïve control group (median = 10.3 pg/mL) (p = 0.0353) but similar production of this cytokine when compared to endemic control (median = 48.9 pg/mL) and *P. vivax*-treated (median = 43.7 pg/mL) groups (Fig. 2c). No significant differences in the TGF-β production was observed for the paired analysis comparing the cytokine level before and after treatment (p = 0.7344).

### IL-27 in vivax malaria

**Fig. 3:**
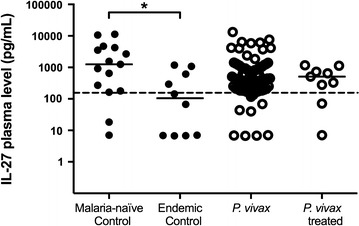
Plasma levels of IL-27 during vivax malaria. Comparative analysis of IL-27 among malaria-naïve control group (n = 15), endemic control group (n = 10), *P. vivax* group (n = 75) and *P. vivax*-treated group (n = 10) were performed using Kruskal–Wallis test followed by Dunn Post-hoc. A p value <0.05 (*) was considered significant. The *dotted lines* (—) represent the detection limit of the assay

IL-27 is a pleiotropic cytokine that can induce either a pro-inflammatory or immunoregulatory response. The production of IL-27 was reduced in individuals living in endemic areas (endemic control, *P. vivax*-infected and *P. vivax*-treated individuals). However, significant differences were observed only between the malaria-naïve control (median = 1257.0 pg/mL) and endemic control (median = 105.3 pg/mL) groups (p = 0.0389, Fig. 3).

### *Plasmodium vivax* infection induced higher levels of MCP-1/CCL2 and IP-10/CXCL10 and lower levels of RANTES/CCL5

**Fig. 4:**
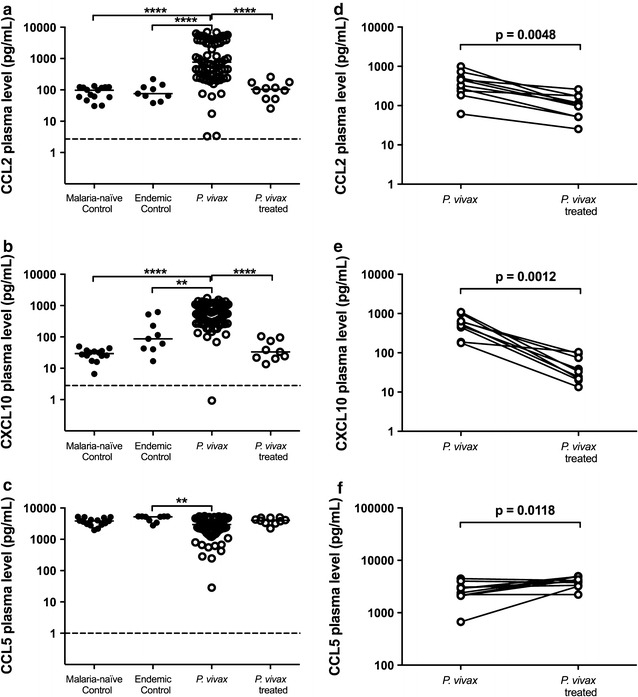
Chemokine plasma levels. Comparative analysis of MCP-1/CCL2 (**a**), IP-10/CXCL10 (**b**), and RANTES/CCL5 (**c**) production among malaria-naïve control group (n = 15), endemic control group (n = 10), *P. vivax* group (n = 75) and *P. vivax*-treated group (n = 10) were performed using Kruskal–Wallis test followed by Dunn Post-hoc. The *dotted lines* (—) represent the detection limit of the assay. Levels of MCP-1/CCL2 (D), IP-10/CXCL10 (**e**) and RANTES/CCL5 (**f**) in acute-phase and convalescence period from *P. vivax* infected patients were determined from *P. vivax* group (n = 10) and *P. vivax*-treated group using Paired *t*-test or Wilcoxon test, according to data distribution. A p value <0.05 was considered significant. *p < 0.05, **p < 0.01, ***p < 0.001, ****p < 0.0001

The plasma levels of MCP-1/CCL2 were increased in *P. vivax*-infected individuals (median = 770.6 pg/mL) when compared to malaria-naïve control group (median = 97.3 pg/mL) and endemic control group (median = 76.0 pg/mL) (Fig. 4a), with further re-establishment to basal levels after treatment (median = 104.5 pg/mL) (p < 0.0001). Plasma samples from *P. vivax*-infected donors also presented higher levels of IP-10/CXCL10 in comparison to control groups (p < 0.0001) but, similarly to MCP-1/CCL2, the IP-10/CXCL10 plasma level in treated individuals presented a baseline production that is equivalent to control individuals (p < 0.001) (Fig. 4b). On the other hand, lower RANTES/CCL5 plasma levels were observed in individuals with *P. vivax* infection when compared to endemic control group (p = 0.0010) (Fig. 4c).

The comparison between plasma levels for both MCP-1/CCL2 and IP-10/CXCL10, before and after treatment (paired analysis), also showed significant differences (p = 0.0048 and p = 0.0012, respectively) (Fig. 4d, e). Despite the absence of significant result between *P. vivax* group and *P. vivax*-treated group due probably to high variability of chemokine levels and differences of sample number, a significant increase was observed in the plasma level of RANTES/CCL5 in the paired t-test after the treatment when following the same individuals (Fig. 4f).

### IL-10 plasma levels were associated with parasitaemia and number of previous malaria episodes

**Fig. 5:**
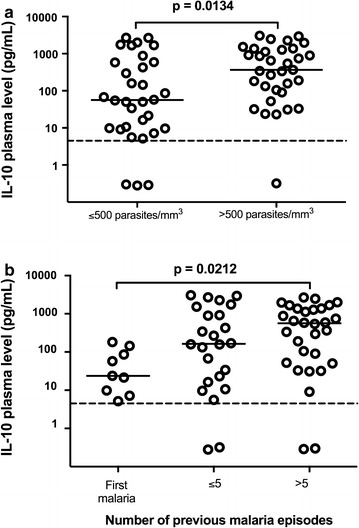
IL-10 plasma levels and their associations. **a** Parasitaemia: high vs low number of parasites. Comparison between high (>500 parasites/cu mm, n = 33) and low (≤500 parasites/cu mm, n = 32) number of parasites was performed using Mann–Whitney test. **b** Number of previous malaria episodes. Comparisons among first malaria (n = 10), ≤5 episodes (n = 24) and >5 episodes (n = 32) were performed using Kruskal–Wallis test followed by Dunn Post-hoc. A p value <0.05 was considered significant. *p < 0.05. The *dotted lines* (—) represent the detection limit of the assay

*Plasmodium vivax*-infected patients were separated into two groups according to parasitaemia: ≤500 parasites/cu mm denominated as low parasitaemia and >501 parasites/cu mm as high parasitaemia (Fig. 5a). Patients with low parasitaemia presented reduced levels of IL-10 (median = 56.2 pg/mL) in comparison to patients with high parasitaemia (median = 366.1 pg/mL) (p < 0.0134). Furthermore, higher number of previous malaria episodes was associated with higher IL-10 plasma levels (p < 0.0242) (Fig. 5b).

### Immune response mediators established a complex network during *Plasmodium vivax* infection

**Fig. 6:**
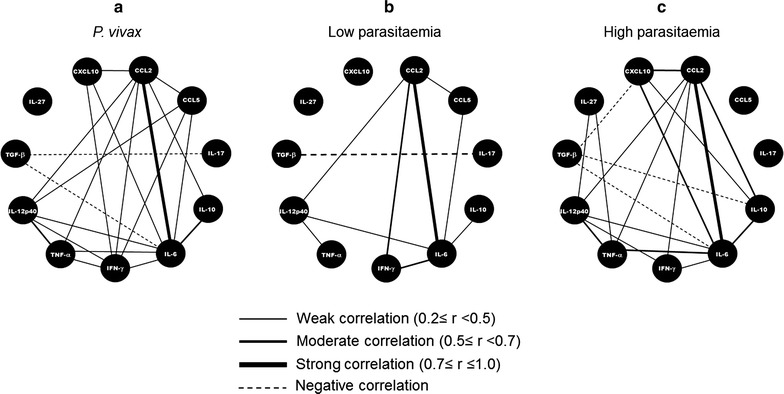
Plasma mediators network. Correlations among 11 mediators during *P. vivax* infection were *plotted* in network graphs. Each *circle* represents a cytokine or chemokine and the connecting lines represent significant correlations between two mediators. *Solid* and *dotted lines*, respectively, represent positive and negative correlations. The *line thickness* represents the significance degree. **a** *P. vivax* group (n = 73). **b** *P. vivax* sub-group with low parasitaemia (≤500 parasites/cu mm) (n = 34). **c** *P. vivax* sub-group with high parasitaemia (> 500 parasites/cu mm) (n = 34). The correlation analyses were evaluated by Spearman correlation test. A p value <0.05 was considered significant

During *P. vivax* infection, the triggering of several plasma mediators might result in a complex interaction network, which may render a weak, moderate or strongly correlation among themselves. The network profiles observed in the *P. vivax* group, as well as the sub-groups classified according to the parasite load, are shown in Fig. 6. Interestingly, high parasitaemia network includes practically all correlation observed in the network of all infected patients (Fig. 6a) highlighting the importance of parasite numbers to host immune stimulation. The correlation network between plasma mediators is less connected in low (Fig. 6b) than high parasitaemia (Fig 6c).

*Plasmodium vivax*-infected patients presented a strong correlation between IL-6 and MCP-1/CCL2 (r = 0.82, p < 0.0001). Furthermore, moderate correlations were observed between IL-6 and IL-10 (r = 0.60, p < 0.0001) and between IL-12p40 and TNF-a (r = 0.54, p < 0.0001). Several weak correlations were observed among other cytokines and chemokines (Fig. 6a). *Plasmodium vivax* patients were further separated into two sub-groups according to parasitaemia: low (≤500 parasites/cu mm) and high (>501 parasites/cu mm). Patients with low parasitaemia presented moderate/strong correlations among inflammatory mediators (IL-6/IFN-γand MCP-1/CCL2). On the other hand, only a weak correlation between IL-6/IL-10 and IL-12p40/TNF was observed in this sub-population (Fig. 6b). Furthermore, patients with high parasitaemia presented moderate correlations between IL-6 and IP-10/CXCL10 (r = 0.56, p < 0.001), IL-10 and MCP-1/CCL2 (r = 0.62, p < 0.001), MCP-1/CCL2 and IP-10/CXCL10 (r = 0.55, p < 0.001) and IL-6/TNF (r = 0.55, p < 0.001) (Fig. 6c).

## Discussion

While some studies have investigated cytokines/chemokines plasma levels during *P. vivax* infection [7–15, 17–19], there is still no agreement regarding the production of cytokines/chemokines and protection. It is well established that a pro-inflammatory response is required for parasite elimination, but an immunomodulatory response is also needed to prevent immunopathology [6]. In the present study, it was shown that *P. vivax* infection induced increased levels of IL-6 and, after treatment, the plasma levels were restored. These findings were in agreement with previous studies during acute phase [11, 15, 17, 18] although the controversial data in the literature with reduction [10, 14] or increase [11] of IL-6 after the treatment, which might be attributed to convalescence period range (7–45 days) in the different studies [8, 10, 11, 13, 14].

Higher levels of some pro-inflammatory mediators such as IL-6, MCP-1/CCL2 and IP-10/CXCL10 were observed in *P. vivax*-infected patients, which were re-established after anti-malarial treatment, suggesting that the parasite infection triggered an inflammatory response. Concomitantly, it seems to be a consensus that *P. vivax* infection induces higher levels of IL-10 during acute phase of vivax malaria [7, 8, 10–13, 15, 17–19] and that the cytokine levels were restored to baseline after treatment [8, 10, 11, 14]. Indeed, a previous study demonstrated that CD4^+^CD25^+^ T cells producing IL-10 play a significant role during *Plasmodium* infection, possibly controlling the pro-inflammatory cytokines IFN-γ and TNF [6]. Of note, previous studies also demonstrated the increased number of circulating Treg cells (CD4^+^CD25^+^Foxp3^+^) during *P. vivax* infection [20, 21]. Regarding the association between IL-10 plasma levels with parasitaemia and number of previous malaria episodes, in the present study, it was observed higher IL-10 plasma levels in patients with high parasitaemia (>501 parasites/cu mm) and with more than five previous malaria episodes. Similar result was observed for the association with parasitaemia, but not with number of previous malaria episodes [18]. The significance of IL-6 and IL-10 has been highlighted during vivax malaria. High levels of these cytokines were observed in uncomplicated cases [18, 22], while low IL-6 levels were observed in patients with complicated malaria [22]. In addition, a positive correlation between IL-6 and IL-10 has also been observed in *P. vivax* infection [10, 18, 22]. Likewise, in the present study correlation analysis between IL-6 and IL-10 demonstrated a positive correlation (r = 0.60) in *P. vivax* group, suggesting the acquisition of a more immunomodulatory profile. The association between IL-10 plasma levels and high parasitaemia could reflect a self-regulation mechanism to protect the excessive inflammatory response because of high antigen stimulation.

During acute episode of vivax malaria, several studies have reported high plasma levels of IFN-γ [7, 10, 12, 15, 19]. In the present study, IFN-γ plasma levels were only observed in 20% of *P. vivax* patient samples. On the other hand, the majority of patients of this group presented higher production of IL-17. However, reports on IL-17 production in the human malarial infection are poorly described [9, 11] and further investigations are required. It is important to highlight that IL-17 may be produced by macrophages, dendritic cells, NK, NKT, γdT cells, CD8^+^ and Th17 [23, 24] and the source of this cytokine during *P. vivax* infection needs more investigation. A previous study showed that CD4^+^ T cells producing IL-17 were increased during vivax malaria [25]. The higher plasma levels of TGF-β and IL-6 in the acute phase could suggest the induction of IL-17, since naïve CD4^+^ T cells require stimulation by IL-6 and TGF-β, and possibly IL-1b, to differentiate in Th17 and to secrete IL-17 [23, 26, 27]. Th17 cells induced by IL-6 and TGF-β also produce IL-10, presenting a regulatory function [28]. It is important to highlight that the low levels of IFN-γ observed in *P. vivax* group could contribute to high levels of IL-17 once IFN-γ negatively regulates the generation of IL-17-producing cells [29].

Despite the high TGF-β plasma levels observed during *P. vivax* infection, there was no consensus about these findings [8, 10, 15]. TGF-β is a potent inductor of Treg cells [30], which may have contributed to the high IL-10 plasma levels observed. Although high IL-17 plasma levels were observed in *P. vivax* group, only a weak negative correlation was detected between IL-17 and TGF-β by the network analysis approach. This connection was also found in low parasite load sub-group. Therefore, the IL-17 and TGF-β roles during *P. vivax* infection require additional investigations. Regarding IL-12p40 and TNF, no significant results were observed in the present study. However, differences in literature findings occur for both cytokines in acute phase [7, 8, 10–13, 15, 17–19] and convalescence period [8, 10, 11, 13, 14].

IL-27 is a pleiotropic cytokine with both pro- and anti-inflammatory actions. This cytokine is a potent inhibitor of Th17 cell development and of IL-17 induction [31, 32]. In the present study, low IL-27 levels were observed in endemic control group compared to malaria-naïve control group. However, no difference was observed regarding to IL-17 between the control groups. In children infected with *P. falciparum*, IL-27 plasma levels were decreased during uncomplicated malaria in comparison to endemic control group. This reduction was more accentuated in severe cases [33]. Thus, the IL-27 role in vivax malaria needs further investigation.

MCP-1/CCL2, known as Monocyte Chemoattractant Protein-1, is a powerful attractant of monocytes, T cells and dendritic cells to inflammatory sites. This chemokine is produced by several cell types, such as epithelial, endothelial, smooth muscle, fibroblasts, astrocytes, monocytes, and microglial cells, and can be induced by TNF, IL-1 and endotoxins [34]. In the present study, high plasma levels of MCP-1/CCL2 were observed during *P. vivax* infection. Similar result was previous described during *P. vivax* infection [7]. The pro-inflammatory IL-6 cytokine induced the mRNA expression and MCP-1 secretion by peripheral blood mononuclear cells [35]. During blood stage of *P. vivax* infection both IL-6 and MCP-1/CCL2 were significantly elevated. The network analysis revealed a strong correlation between IL-6 and MCP-1/CCL2, regardless of parasite load, suggesting that these mediators were induced by the infection itself. These findings support the hypothesis that IL-6 and MCP-1/CCL2 pathway plays a central role in response to *P. vivax* infection. Interestingly, the network analyses for the high and low parasitaemia groups have shown that individuals with high parasitaemia exhibit moderate/strong correlation between IL-6/IL-10 and MCP-1/CCL2. However, patients with low parasitaemia exhibited weak correlation between IL-6 and IL-10, losing the interaction between IL-10 andMCP-1/CCL2. These data associated with the positive correlation between IL-6/IFN-γ reinforce the significance of the IL-6/MCP-1/IFN-γ axis in controlling parasitaemia, which could contribute to the lower parasite load observed.

IP-10/CXCL10 (IFN-inducible protein 10) is another chemokine that is induced by IFN-γ [36] as well as by IL-17 [37] in different cell types. This chemokine is involved in inflammatory processes, being capable of attracting macrophages, dendritic cells, NK cells and activated CD4^+^ and CD8^+^ T cells towards inflamed tissues [36]. In the present study, *P. vivax* infected patients presented elevated IP-10/CXCL10 plasma levels, but only weak positive correlation with IFN-γ. No correlation was observed between IP-10/CXCL10 and IL-17, although both mediators were elevated during *P. vivax* infection. However, when patients were separated according to the parasite load, this connection was lost, and moderate correlation was established between IP-10/CXCL10 and IL-6, only in patients with high parasitaemia. In *P. falciparum* infection, IP-10/CXCL10 has been identified as biomarker (in serum and cerebrospinal fluid) associated with elevated risk of fatal cerebral malaria [38]. On the other hand, higher IP-10/CXCL10 plasma levels, as well as IFN-γ and IL-10, were observed in vivax malaria patients with mild anaemia in comparison to no anaemia [12].

RANTES/CCL5, known as Regulated upon Activation Normal T cell Expressed and Secreted, is an inflammatory chemokine attractant of T cells, basophils, eosinophils, and dendritic cells to inflammatory site [39]. RANTES/CCL5 is produced predominantly by CD8^+^ T cells, epithelial cells, fibroblasts, and platelets [40]. In children infected by falciparum malaria low mRNA and RANTES protein levels were associated with severe malaria [41]. Lower RANTES levels were also found in children with cerebral malaria and a strong positive correlation was verified between RANTES levels and platelets count [42]. In the present study, vivax malaria patients have shown significant low RANTES/CCL5 levels, but just weakly associated with IL-6, IL-12p40, IFN-γ or MCP-1/CCL2. The lower levels of RANTES/CCL5 could be explained by the CD8^+^ T cells reduction [43–45] and thrombocytopaenia [8, 10, 11, 44–46] observed during vivax malaria. A study carried out with children infected with *P. falciparum* observed an association between thrombocytopaenia and lower RANTES plasma levels [47].

## Conclusion

Taken together, the multiple analyses performed in the present study allowed the identification of an immunological signature from plasma mediators associated with *P. vivax* acute infection. IL-6, MCP-1/CCL2 and IL-10 could be recognized as biomarkers of acute phase of *P. vivax* infection. These results provide new insights into the complex relationship among mediators that are triggered during *P. vivax* clinical malaria.
